# Phage Therapy: Combating Infections with Potential for Evolving from Merely a Treatment for Complications to Targeting Diseases

**DOI:** 10.3389/fmicb.2016.01515

**Published:** 2016-09-26

**Authors:** Andrzej Górski, Ryszard Międzybrodzki, Beata Weber-Dąbrowska, Wojciech Fortuna, Sławomir Letkiewicz, Paweł Rogóż, Ewa Jończyk-Matysiak, Krystyna Dąbrowska, Joanna Majewska, Jan Borysowski

**Affiliations:** ^1^Bacteriophage Laboratory, Ludwik Hirszfeld Institute of Immunology and Experimental Therapy, Polish Academy of Sciences, WroclawPoland; ^2^Phage Therapy Unit, Ludwik Hirszfeld Institute of Immunology and Experimental Therapy, Polish Academy of Sciences, WroclawPoland; ^3^Department of Clinical Immunology, Transplantation Institute, The Medical University of Warsaw, WarsawPoland; ^4^Katowice School of Economics, KatowicePoland

**Keywords:** bacteriophage, phage therapy, antibiotic resistance, anti-phage antibodies, inflammation, reactive oxygen species, compassionate use

## Abstract

Antimicrobial resistance is considered to be one of the greatest challenges of medicine and our civilization. Lack of progress in developing new anti-bacterial agents has greatly revived interest in using phage therapy to combat antibiotic-resistant infections. Although a number of clinical trials are underway and more are planned, the realistic perspective of registration of phage preparations and their entering the health market and significantly contributing to the current antimicrobial crisis is rather remote. Therefore, in addition to planning further clinical trials, our present approach of phage treatment carried out as experimental therapy (compassionate use) should be expanded to address the growing and urgent needs of increasing cohorts of patients for whom no alternative treatment is currently available. During the past 11 years of our phage therapy center’s operation, we have obtained relevant clinical and laboratory data which not only confirm the safety of the therapy but also provide important information shedding more light on many aspects of the therapy, contributing to its optimization and allowing for construction of the most appropriate clinical trials. New data on phage biology and interactions with the immune system suggest that in the future phage therapy may evolve from dealing with complications to targeting diseases. However, further studies are necessary to confirm this promising trend.

## Is it Ethical to Continue Not Pursuing Phage Therapy? (Henein, 2013)

On May 19, 2016 a review on antimicrobial resistance (AMR) commissioned by the UK Prime Minister was published. AMR – considered to be a challenge to health and our entire civilization – compared to terrorism and global warming – costs some 700,000 deaths annually and, if not controlled, would lead to 10 million deaths by 2050, exceeding the toll of cancer. To prevent medicine being cast back to the dark ages, the review suggests a number of actions promoting alternatives to drugs. Interestingly, phage therapy has been placed at the top of a table presenting possible alternative products to tackle infections (O’Neill, 2016).

Earlier this year another milestone document, also a review delivered by 24 scientists from academia and industry and commissioned by the Wellcome Trust, aimed at identifying the prospective therapeutic replacements for antibiotics, considered a number of key factors in this issue: (1) feasibility of informative clinical trials, (2) magnitude of medical potential, (3) likelihood and consequences of resistance, (4) level of current research activity, (5) likely time to registration, and (6) activities enabling validation and progression. Again, among the top ten approaches which the group considered merited attention, phage therapy was included, with earliest anticipated registration as of 2022 (Czaplewski et al., 2016).

The recent discovery of a plasmid-borne colistin resistance gene heralds the emergence of truly pan-drug resistant bacteria (McGann et al., 2016) and suggests that the competition between the prospect of a pre-antibiotic era and registration of phages as available medicinal products may be won by bacteria, with all the dramatic consequences for our civilization emphasized by the report mentioned above. This strongly suggests that our model of phage therapy applied as experimental treatment should continue to be implemented by other medical centers, in line with the recent suggestions. As Gill and Young have pointed out: “…we can see no compelling reason why phage therapy cannot be made more widely available on compassionate use (CU) grounds to patients aﬄicted with serious bacterial infections which are refractory to standard treatments. A Hirszfeld Institute model for such treatment could be implemented. As in Poland, the availability of such a treatment would not only save lives but allow for the collection of clinical data under well-documented clinical conditions within the USA” (Gill and Young, 2011). In a more recent article in *Science*, the authors recommend that: “major hospitals should establish validated phage collections for CU applications, where, in many instances, the bacterial pathogen has been identified and could be tested for sensitivity to a library of phages” (Young and Gill, 2015). This assumption was also fully supported by other authors, who have emphasized that introducing phage therapy into Western clinical practice in a collaborative, CU fashion would not require further deviation from the current standard of care (Kutter et al., 2015). What is more, our approach does not constitute market placement and practically places production of phage preparations for our patients use outside of the scope of European Medicinal Product Directive 2001/83/EC which has recently been fully confirmed by legal analysis reported by Verbeken et al. (2014, 2016). “The debate is no longer about the pros and cons of phage therapy, but rather about how we can move forward for patients to benefit from this therapy. We are proposing to set up dedicated public structures, National Reference Centers (NRC) for bacteriophage therapy. These NRC will pilot these treatments and put in place production of hospital-based bacteriophage solutions, and application protocols that will ensure adequate product quality, patient safety and monitoring of treatment efficacy” (Debarbieux et al., 2016). We could not agree more, as we have done so when our phage therapy center was established in 2005, and have been continuing our activities that have made it possible to gain invaluable experience derived from patient care and careful monitoring clinical, laboratory and immunological indices (Górski et al., 2012; Międzybrodzki et al., 2012). The establishment of our center and earlier activities of our Institute were facilitated by a 100 years rich experience of former Soviet Union Countries (especially Georgia and Russian Federation) covered by in-depth excellent reviews (Chanishvili et al., 2009; Kutateladze and Adamia, 2010; Chanishvili, 2016).

## Clinical Trials Vs. Observational Studies and Experimental Therapy: Their Role in Further Advancement of Phage Therapy

One should also be aware that – in addition to randomized clinical trials (RCT) – observational studies are an important category of study designs, considered to be the next best investigational method which may even yield comparable results (Benson and Hartz, 2000; Concato et al., 2000). One should cite here FDA Commissioner who has pointed out: “Although randomized trials perform an essential role in the development of therapies, we should not neglect the crucial and complementary role that can be played by high-quality observational studies… However, truly effective use of this volume of observational data will require considerable methodological development, including whether an observational study can provide sufficient evidence to render a randomized trial unnecessary” (Califf and Ostroff, 2015). In this context it is noteworthy that the use of unapproved drugs outside of formal clinical trials in patients posing difficult clinical dilemmas has been accepted in a variety of countries including the US, Canada, Australia, many European countries such as the UK, Germany, Austria, Switzerland, France, Italy, as well as Japan and China. Such treatment is most often called CU or expanded access (EA), and its basic rules vary by country, most often encompassing chronic, seriously debilitating or life-threatening disease, lack of effective approved drugs for use in a given patient, approval by an ethics committee, and informed consent. CU may involve the use of a therapeutic product at any stage of its development, including preclinical and early phases of clinical trials (Bedell, 2010; Whitfield et al., 2010; Walker et al., 2014). Thus, CU of phage preparations may be considered an alternative to phage therapy performed using preparations that would have been formally approved following successful completion of clinical trials.

Recently, a number of review articles covering various issues related to phage therapy (including completed and ongoing clinical trials, and regulatory and ethical issues at national, EMA and FDA levels) have been published; therefore those aspects will not be addressed here. Suffice it to say that the first randomized double blind, placebo-controlled phase I/II clinical trial on patients with otitis externa reported symptom amelioration and a decrease of mean *Pseudomonas aeruginosa* titers in those patients associated with significant phage replication *in vivo* (a 200-fold titer increase), as well as a lack of adverse effects. However, a recently completed phase II trial in children with acute *Escherichia coli* diarrhea did not show superiority over the current standard of care. The causes of this clinical trial’s failure are obscure at the moment; increased levels of *Streptococcus* in those children raise fundamental questions on the causative agent(s); in addition, the relatively short duration of phage administration (4 days) might also have contributed to the unsatisfactory results (Sarker and Brussow, 2016). In 2017 the current Phagoburn clinical trial evaluating phage therapy in the treatment of burn wounds infected with *E. coli* and *P. aeruginosa* should be completed, while Technophage has received FDA clearance to begin a trial of a phage cocktail for the treatment of infected chronic ulcers occurring in diabetic foot infections. New data on the efficacy of phage therapy are therefore on the horizon; however, new clinical trials addressing important clinical dilemmas (e.g., urinary tract infections) are urgently needed (Chan et al., 2013; Ly-Chatain, 2014; Letkiewicz, 2015; Pirnay et al., 2015; Vandenheuvel et al., 2015; Young and Gill, 2015; Chanishvili, 2016; Debarbieux et al., 2016; Expert round table on acceptance and re-implementation of bacteriophage therapy, 2016; Verbeken et al., 2016).

Our phage therapy unit has been accepting patients since 2005, so during the past 11 years, we have gained rich practical experience resulting from direct patient care and monitoring their clinical, laboratory and immunological parameters. Those observations were summarized in our report published 4 years ago (Międzybrodzki et al., 2012). In the subsequent years, we have analyzed the data derived from more than additional 150 patients, which essentially confirmed our earlier report, both with regard to the therapy efficacy (good results achieved in approximately 40% of cases). Most importantly, we have re-confirmed the safety of the therapy and indicated a minimal number of cases in which it should have been discontinued due to side effects. The fact that immunocompetence in patients with depressed immunity has been evaluated as antibody responses to phage administered by the intravenous (iv) route as well as historical data indicating that patients were treated using that mode of phage administration with outstanding efficacy and safety support our data and suggest that iv phage therapy using purified preparations should be seriously considered (Chanishvili, 2016; Speck and Smithyman, 2016).

Although current protocols of phage therapy carried out at our institute have not supplied formal proof of phage therapy efficacy – according to the requirements of evidence-based medicine – they nevertheless provided valuable clinical and laboratory data suggesting the optimal pathways for clinical trials as well as novel and interesting data on phage interactions with the immune system. For example, already 10 years ago, we described successful eradication of MRSA intestinal carrier status with subsequent eradication of genitourinary tract infection with the same pathogen (Leszczyński et al., 2006); this was later confirmed by good results of urinary tract infections using oral administration of bacteriophage preparations (Międzybrodzki et al., 2012). Recently, Galtier et al. (2016) from the Pasteur Institute using a mouse model and oral phage administration showed 1000-fold titer reduction of uropathogenic *E. coli*. As pointed out (Brussow, 2016), this could lead to successful application of phage against intestinal and by extension urinary pathogens. Our data strongly suggest that at least some forms of urinary tract infections can be treated with oral phage preparations targeting causative pathogens.

Studies *in vitro* and in experimental animals suggest that the use of phage cocktails may be superior to monotherapy with a single phage targeting a given pathogen because of a generally wider target range. In addition, the likelihood of the development of phage resistance in bacteria is much less likely using cocktails than single phages (Chan et al., 2013; Vandenheuvel et al., 2015). However, some of our therapeutic phages are sufficiently polyvalent as to cover more than 60% of target strains. Moreover, as emphasized earlier, we have achieved an approximately 40% rate of good results including approximately 20% eradication rate using phage monotherapy although the phage resistance phenomenon was occurring in the course of treatment (Międzybrodzki et al., 2012). Furthermore, our preliminary observations do not suggest clear clinical superiority of cocktails vs. monovalent phage preparations This finding is in agreement with the data of Brown et al. (2016) who showed that the use of a phage cocktail was not superior to using a single phage. Of note, high antiphage activity of sera was observed in 43% of patients treated locally with cocktails, in contrast to only 17% of patients on phage monotherapy, which suggests that antibody responses to phage therapy may vary depending on whether patients receive monotherapy or a cocktail of phages. In fact, phages may induce different levels of immunization, and antibody responses to some phages contained in cocktails may be exceptionally high; this suggests that such phages present in cocktails may induce adjuvant-like effects (Łusiak-Szelachowska et al., 2016). One should also keep in mind that the registration of a multicomponent phage cocktail should be considerably more complex than a monovalent preparation, as experienced by Pherecydes which was requested to demonstrate stability of all components of their elaborate cocktail (Servick, 2016).

As pointed out, our experience suggests that despite bacteriophage-resistant strain proliferation, phage therapy may be successful, which may depend on virulence reduction in such bacterial strains (lower growth rate, underexpression of virulence genes, loss of pathogen’s ability to attach to human cells, markedly reduced lifespan (Leon and Bastias, 2015). Without the pressure of phages, resistant strains may revert to the parental phenotype or may be displaced by non-resistant virulent strains which may explain why prolonged phage therapy may be sometimes more efficacious than a shorter protocol. Our clinical data are confirmed by others (Capparelli et al., 2010) who demonstrated in mice that phage-resistant bacteria may not only be avirulent, but are also rapidly cleared by the immune system and, importantly, induce a balanced anti-inflammatory response (repression of transcription of the TNF-α and IFN-γ genes and induction of expression of the IL-4 and IL-6 genes). What is more, acquired phage resistance may be associated with greater sensitivity to antibiotics. Those important data strongly suggest that – from the clinical point of view – the development of phage resistance by relevant pathogens should not always be considered as an undesired phenomenon, as it may cause offending bacteria to become increasingly antibiotic-sensitive and allow for renewed use of historically effective antibiotics that have been rendered useless by the evolution of antibiotic resistance. This approach has the potential to extend the effective lifetime of antibiotics in our drug arsenal and broaden the spectrum of those drugs, greatly reducing the burden on drugs of the last resort, preserving them for future use (Chan et al., 2013). The issue of combined use of phages and antibiotics is of evident clinical significance. Some experimental data *in vitro* and in experimental animals may suggest that such treatment could be superior to phages or antibiotics alone; this problem has recently been discussed in some detail (Torres-Barcelo and Hochberg, 2016) and therefore will not be elaborated here. Also, one can find some data in patients suggesting higher efficacy of such combined therapy (Kutateladze and Adamia, 2010; Chanishvili, 2016). Most proponents of this treatment highlight its potential. However, as rightly pointed out (Torres-Barcelo and Hochberg, 2016), there are also potential drawbacks of phage-antibiotic combinations such as the development of double-resistant variants, similar to the effects of antibiotic cocktails, which could have catastrophic consequences not only for patients thus treated but for further prospects of successful combat of AMR. Our policy has been to add antibiotics to phage treatment solely in polyinfections in which no phage was available to match an additional pathogen. Evidently, planned clinical trials involving phage therapy should also include combination treatment with antibiotics to provide more reliable data on this important clinical dilemma.

## Anti-Inflammatory Effects of Phages

One of the most promising aspects of phage therapy is its remarkable anti-inflammatory action. We have noted a significant decrease in mean C reactive protein (CRP) values and leukocyte counts, with a similar tendency of erythrocyte sedimentation rate (Międzybrodzki et al., 2009). In some patients the reaction of CRP was dramatic and decreased from 50 to 5 mg/l within 2–3 weeks of the treatment even though complete eradication of infection was not achieved. This suggests that phage can exert its anti-inflammatory action by at least two mechanisms: one dependent on its well-known anti-bacterial action, and another which acts directly on phenomena responsible for the development of inflammatory processes. In fact, we have demonstrated that phage can diminish cellular infiltration of allogeneic skin transplants in mice and activation of the nuclear transcription factor NF-kappa B (which leads to expression of proinflammatory cytokines, chemokines, and adhesion molecules (Górski et al., 2006a). A short tail fiber protein, tail adhesion gp12, mediates adsorption of T4 phage to *E. coli*, binding LPS. Recently, our group demonstrated that recombinant gp12 counteracts proinflammatory effects of LPS *in vivo*, causing the reduction of serum IL-1 and IL-6 levels as well as a decrease of inflammatory infiltration in spleen and liver (Miernikiewicz et al., 2016). What is more, we have observed that phages and their surface proteins do not stimulate inflammatory mediator and reactive oxygen species (ROS) production when administered to mice (Miernikiewicz et al., 2013). This confirms and extends our earlier reports indicating that T4 phage lysates and its purified preparations induce only minimal levels of respiratory burst in whole blood monocytes and neutrophils while staphylococcal phage preparations do not stimulate the production of ROS at all (Borysowski et al., 2010). Furthermore, phages diminish ROS production induced by bacteria and endotoxin (Międzybrodzki et al., 2008), which highlights their potential in the treatment of sepsis (Weber-Dąbrowska et al., 2003). As shown by our group, phages do not induce granulocyte degranulation (Borysowski et al., submitted for publication), their administration to patients is not associated with leukocytosis (conversely, as stated earlier, they may reduce the number of circulating leukocytes in patients with bacterial infection; it is also noteworthy that phage therapy does not cause eosinophilia) (Międzybrodzki et al., 2012). In conclusion, our studies performed *in vitro* as well as *in vivo* in experimental animals and patients strongly suggest that phages may exert anti-inflammatory effects that can be useful clinically. The success of phage therapy depends on the ability of phages to migrate to infected tissues and achieve concentrations necessary to eradicate infection and exert anti-inflammatory action. In this regard, we proposed to engineer phages armed with tissue-specific peptides – this methodology, not necessarily involving genetic manipulations, may significantly enhance the effectiveness of phage therapy (Górski et al., 2015).

## Phages and the Immune System

Górski and Weber-Dąbrowska (2005) proposed that phages may mediate immunomodulatory, probiotic-like functions and this phenomenon could be relevant in regulating local immunity in the intestinal tract where phages are an abundant part of the microbiome. What is more, phage translocation from the intestines might contribute to phages mediating such probiotic-like functions also in other parts of the body (Górski et al., 2006b). As the evidence on phage engagement in regulating immunity is accumulating, those data add credence to our hypothesis. In fact, our comprehensive review addressing the issue of phage interactions with the immune system and their possible practical implications, especially in relation to the therapy, highlights the potential role of phages as clinically useful immunomodulators. Our data indicate that about half the patients prior to phage therapy are immunodeficient; although the therapy may cause some fluctuations of immune parameters, its beneficial effects are not correlated with upgraded immunity (except for an increase of phagocytosis noted in some patients which also appears to have some positive prognostic value). Therefore, the therapeutic effects of phage therapy are associated with its anti-bacterial and anti-inflammatory action rather than resulting from correction of depressed immunity, so the potential vaccine-like effect of phages is not responsible for their curative activity (Górski et al., 2012). Our data on monitoring phagocytosis by neutrophils and monocytes of patients on phage therapy suggest that the therapy may instead correct existing deficiencies in phagocyte functions (see above). In fact, we have shown that phages – both *in vitro* and *in vivo* – do not adversely affect the ability of phagocytes to kill bacteria – both standard strains as well as specific pathogens isolated from patients (Jończyk-Matysiak et al., 2015). Also, phages do not impair migratory activity of human phagocytes *in vitro* (Kurzępa, 2011), whereas they may markedly diminish tissue infiltration with those cells at the foci of inflammation (Górski et al., 2006a; Miernikiewicz et al., 2016).

An important clinical dilemma is whether phage therapy may be safe and efficient in immunosuppressed host (Borysowski and Górski, 2008). Our data obtained in patients with antibiotic-resistant infections who frequently have associated immunodeficiency confirm the value of the therapy in this syndrome. Also, phages have been successfully applied in cancer patients and renal allograft recipients (Borysowski and Górski, 2008). Those clinical observations were also confirmed experimentally by our group (Zimecki et al., 2010), who described the protective effects of phage therapy in immunodeficient mice subjected to myeloablative and immunosuppressive conditioning followed by bone marrow transplant and infected by sublethal and lethal dose of *Staphylococcus aureus*. Of note, we have shown that phage preparations do not enhance inflammatory processes in experimentally induced autoimmune disease in mice and may even have protective and therapeutic action (Międzybrodzki et al., unpublished data). These findings may suggest that phage therapy is also safe in patients with autoimmune disorders. If confirmed, those data should be especially relevant as such patients are especially prone to multidrug resistant infections.

## Antibody Responses to Phage Therapy

Animal models allow for comprehensive studies of immune responses to bacteriophages *in vivo*. Induction of specific anti-phage antibodies has probably been the most extensively studied in mice, resulting in a multi-faceted description of this phenomenon. This comprises dose, schedule and route of administration effects, with regard to primary classes of immunoglobulins, and with reference to various immunogenicity of particular structural elements of bacteriophages.

A model bacteriophage T4 has been demonstrated to be able to induce specific antibody both after phage injection into peritoneum (Dąbrowska et al., 2014) and in long-term *per os* treatment (Majewska et al., 2015). In the experimental model of *per os* treatment, efficient induction of specific antibody production required long exposure of animals to the phage. The authors observed a significant increase in serum IgG on day 36. Once the IgG level reached a peak, it remained high throughout the experiment, even after the phage was removed from the diet. It did not, however, impact gastrointestinal phage transit. Interestingly, no clear IgM peak preceded the IgG boost, which is in contrast to immunization by parenteral applications, where a significant increase of IgM has been reported (Dąbrowska et al., 2014; Hodyra-Stefaniak et al., 2015).

A characteristic feature of oral administration is the secretory IgA production in the gut. In fact, this can also be observed as a result of bacteriophage administration *per os*, but again its induction requires long and high dose exposure to bacteriophage. An increase in secretory IgA was reported on day 79 of the oral treatment with T4. Importantly, the increase of phage-specific IgA in the gut correlated with the lack of viable phage particles detected in the feces. Thus, specific anti-phage IgA can be considered as a factor limiting phage viability in the gut (Majewska et al., 2015).

In order to draw a general conclusion about T4 phage immunogenicity for the needs of therapeutic approaches, an estimated adequate dose in humans was calculated: 2 × 10^10^ pfu per mouse corresponds to 7 × 10^13^ pfu per human patient daily (using a simplification of volumes as proportional to weight across species). The dosage used in therapeutic approaches in humans is usually much lower: in the 20th century, tablet or liquid formulations that were used in oral treatment of humans contained 10^5^ to 10^11^ pfu/dose (Sulakvelidze et al., 2001), while daily therapeutic phage doses used in the Phage Therapy Unit of the Institute of Immunology and Experimental Therapy (IIET) in the years 2008–2010 ranged between 3 × 10^7^ and 6 × 10^10^pfu per patient (Międzybrodzki et al., 2012). Also in T4 phage safety tests conducted by Bruttin and Brüssow (2005) the total amount of phage preparations administered to human volunteers was much lower and equaled 9 × 10^7^ pfu; no specific antibodies were detected following the safety tests. Taking into consideration the unusually high dosage and the time of continuous treatment that was necessary to elicit a humoral response (2 weeks in the case of IgG and as long as 2 months in the case of IgA), T4 phage immunogenicity in oral administration was defined as weak.

Microbiological assessment of bacterial fecal flora during the prolonged feeding of mice with T4 showed no substantial differences between phage-treated and control mice. The emergence of phage-resistant *E. coli* strains was observed in phage-treated mice very late: on day 92 (Majewska et al., 2015). This fact is important in the light of recent studies of the human microbiome that have demonstrated links between dysbiosis in the gut and numerous health problems, both located within the gastrointestinal tract and in other parts of the body (Kau et al., 2011). Animal studies suggests that the impact of orally applied phage on gut natural microflora is minimal.

Structural proteins of bacteriophages may differ in their individual immunogenicity. Furthermore, the route of administration may play a role in the resulting ability of the proteins to induce specific antibodies. T4 phage proteins reported as highly immunogenic when applied intraperitoneally were major capsid protein gp23 and highly antigenic outer capsid protein gpHoc (Dąbrowska et al., 2014). However, oral administration of this phage resulted in immunization mostly to gpHoc and gp12 (tail spike) (Majewska et al., 2015). These data highlight the fact that route of administration plays a role in determining the fate of phage particles in the context of the specific humoral response. Gp12 is crucial in the process of phage adsorption and infection of bacterial host cells; therefore, the humoral response directed toward this particular protein may impair antibacterial properties of the phage and consequently impact the efficacy of phages as therapeutic agents. Hence, further studies aiming to identify the molecular basis for this response may, in the future, facilitate the optimal choice and design of phage use in therapy. On the other hand, a high-level humoral response may turn out to be beneficial in some scenarios. T4 phage has been effectively used as a phage display platform for foreign antigens, often displayed as Hoc fusion proteins (Rao group: Jiang et al., 1997; Sathaliyawala et al., 2006; Shivachandra et al., 2006, 2007). In this case, the ability of Hoc protein to induce a high-level humoral response may result in more efficient immunization to the fusion protein, which is highly desirable in vaccine development.

Since bacteriophages can be neutralized by specific antibodies *in vivo* and *in vitro*, a few possible mechanisms of phage inactivation have been considered. The most straightforward one was direct interaction of antibodies with phage proteins that are necessary for infection of bacterial cells; phage cannot attack bacteria since these proteins have been occluded by antibodies (Jerne and Avegno, 1956). However, anti-head immunization has also been demonstrated to reduce phage activity. In this case aggregation of phage particles as well as the antibody-dependent complement pathway were proposed as the mechanisms of phage inactivation (Dąbrowska et al., 2014).

Our recent papers have analyzed the practical issues of neutralizing antibody responses to phages in their clinical context based on the largest patient material ever available (Łusiak-Szelachowska et al., 2014, 2016). As noted, several key factors are responsible for those antibody responses: patients’ immune status, route of phage administration, antigenicity of a given phage, and monotherapy vs. phage cocktails. Furthermore, the clinical significance of the production of anti-phage antibodies is unclear at the moment, as we have not confirmed a correlation between their appearance and therapy outcome. Conversely, good clinical results may be achieved with concurrent high serum neutralizing antibody levels against administered phage. Interestingly, anti-staph phage that effectively controls *S. aureus* growth and reduces bacterial viability both *in vitro* and in a skin infection mouse model loses its killing effect when the phage is cultured in the presence of human blood (Pincus et al., 2015). It may well be that local antibody interactions with phage at the foci of infection are more relevant for therapy success than serum antibody levels, whose raised values may – paradoxically – at least in some patients indicate a good clinical outcome signaling the recovery of the immune system and its more active participation in clearing infection. Our data suggesting that recovery of phagocytosis may be a good prognostic sign for therapy outcome provide food for thought for such hypothesis.

## Future Prospects of Phage Therapy

It has been highlighted that phages – unlike classical antibiotics – are biological entities of incredible diversity and adaptability and many surprises may be in store (Young and Gill, 2015). The above mentioned data suggest that phages could interfere with some viral and fungal infections which could extend its potential therapeutic value beyond well-known antibacterial action. One could list here our recent data showing that T4 phage can inhibit infection of the target cells by an adenovirus (Przybylski et al., 2015) as well as the data from Stanford indicating that *P. aeruginosa* phage inhibits *Aspergillus* (Penner et al., 2016), which suggest that future applications of phage therapy may extend beyond its well known antibacterial action. In recent years, we have been facing unprecedented growth of interest in the human microbiome which has emerged as an important factor in human physiology and disease including obesity and diabetes, cancer and susceptibility to its chemotherapy, cognition and depression, etc. Therefore, manipulation of the microbiome is currently believed to have great potential for efficient therapy of disorders posing a challenge to medicine and civilization (Blaser, 2014). Phage-mediated immunomodulation of the enteric immune system and microbiome may be an important key to the success of this strategy (Górski et al., 2003, 2006b). One cannot thus exclude that the future phage therapy may be focused on our microbiome. Today, we can use phage therapy to combat urinary tract infections secondary to kidney stones which treats complications rather than a disease itself. Gut microbiota is unique in kidney stone disease (Kelsey, 2016). One could therefore envisage using phages to manipulate gut microbiome to treat and prevent the development of renal stones not only their complications. Moreover, phage ability to reduce the production of ROS and inflammatory processes (e.g., tissue infiltration by leukocytes) could be helpful to ameliorate the clinical course of patients with disorders where those phenomena are relevant (e.g., graft rejection, inflammatory bowel disease, etc.).

While the Canadian doctor F. d’Herelle discovered phages, another Canadian medical authority has emphasized: “The good physician treats the disease, the great physician treats the patient who has the disease” (Sir William Osler). A good example of this philosophy is the recent data suggesting that intensive glucose lowering protocols cause more harm than benefit in patients with type 2 diabetes (The Action to Control Cardiovascular Risk in Diabetes Study Group, 2008). Patients with antibiotic-resistant infections have complex clinical problems and frequently pose difficult clinical dilemmas, resistant infection being an important, but not the sole component of their morbidity. The main purpose of a physician should be to provide optimal therapy considering all aspects of a patient’s disorder, that is to heal the patient, not just eradicate infection (certainly not at any cost). Some reviews on phage therapy appear to disregard this principle; in this regard, one could cite an important recent opinion of Nature Medicine: “researchers need to reach back to the patients, but in a way that steers clear of giving medical advice” (Knoepfler, 2016). Phage therapy has the potential to go beyond from merely treating an infection in a patient to treating its causes and prevent further complications, a promise that requires further studies and confirmation. Tailoring medical treatment to the individual characteristics, needs and preferences of each patient fulfills the promise of a new era of medical product development referred to as personalized (precision) medicine (Food and Drug Administration, 2013). Phage therapy constitutes an excellent example of this novel trend.

## Author Contributions

AG drafted the main part of the manuscript, KD, JM, JB, EJ-M contributed parts of the manuscript, all authors revised the manuscript.

## Conflict of Interest Statement

AG, RM, BW-D, KD, JM, and JB are co-inventors of patents owned by the Institute and covering phage preparations. All other authors declare that the research was conducted in the absence of any commercial or financial relationships that could be construed as a potential conflict of interest.
